# The Obese Liver Environment Mediates Conversion of NK Cells to a Less Cytotoxic ILC1-Like Phenotype

**DOI:** 10.3389/fimmu.2019.02180

**Published:** 2019-09-11

**Authors:** Antonia O. Cuff, Francesca Sillito, Simone Dertschnig, Andrew Hall, Tu Vinh Luong, Ronjon Chakraverty, Victoria Male

**Affiliations:** ^1^Department of Surgery and Cancer, Imperial College London, London, United Kingdom; ^2^Institute of Immunity and Transplantation, University College London, London, United Kingdom; ^3^Institute for Liver and Digestive Health, Royal Free Hospital and University College London, London, United Kingdom

**Keywords:** NK cells, ILC1, obesity, NAFLD, immunometabolism, TGFβ

## Abstract

The liver contains both NK cells and their less cytotoxic relatives, ILC1. Here, we investigate the role of NK cells and ILC1 in the obesity-associated condition, non-alcoholic fatty liver disease (NAFLD). In the livers of mice suffering from NAFLD, NK cells are less able to degranulate, express lower levels of perforin and are less able to kill cancerous target cells than those from healthy animals. This is associated with a decreased ability to kill cancer cells *in vivo*. On the other hand, we find that perforin-deficient mice suffer from less severe NAFLD, suggesting that this reduction in NK cell cytotoxicity may be protective in the obese liver, albeit at the cost of increased susceptibility to cancer. The decrease in cytotoxicity is associated with a shift toward a transcriptional profile characteristic of ILC1, increased expression of the ILC1-associated proteins CD200R1 and CD49a, and an altered metabolic profile mimicking that of ILC1. We show that the conversion of NK cells to this less cytotoxic phenotype is at least partially mediated by TGFβ, which is expressed at high levels in the obese liver. Finally, we show that reduced cytotoxicity is also a feature of NK cells in the livers of human NAFLD patients.

## Introduction

Natural killer (NK) cells are innate lymphoid cells that recognize and kill virally infected and cancerous cells. In mice, NK cells were long defined as Lineage-negative NK1.1^+^. However, it has recently come to light that, in tissues, cells defined in this way contain at least two distinct populations: circulating, or conventional, NK cells that are CD49a^−^CD49b^+^ and tissue-resident NK-like cells that are CD49a^+^CD49b^−^ (1). These have been called either tissue-resident NK cells or innate lymphoid cells, type 1 (ILC1): here, we call them ILC1 (2). A population of cells that is in many ways equivalent to mouse tissue-resident CD49a^+^ NK cells is also present among CD3^−^CD56^+^ cells in human livers and has been proposed to be ILC-like on the basis of its parallels with mouse ILC1 (3). However, CD56^−^ cells that display one of the proposed phenotypes for human ILC1 (4) have also been reported in human liver (5). For this reason, here we refer to the equivalent cells in humans by the descriptive term liver-resident NK cells. The finding that Lin^−^ NK1.1^+^ cells in mouse and CD3^−^CD56^+^ cells in human livers are in fact heterogeneous populations means that it is now necessary to re-examine the roles of these cells, distinguishing between NK and ILC1 or resident subpopulations.

Non-alcoholic fatty liver disease (NAFLD) is a spectrum of disease, usually associated with obesity, in which excess fat builds up in the liver. Simple steatosis can progress to a chronic inflammatory condition known as non-alcoholic steatohepatitis (NASH). This in turn progresses to fibrosis and ultimately cirrhosis, which increases the risk of developing hepatocellular carcinoma. NASH patients have been reported to have increased numbers of CD3^−^CD56^+^CD57^+^ cells, which are likely to represent circulating NK cells, in their livers (6), although a more recent study was unable to replicate this finding (7). The expression of NK cell-activating ligands is also increased in the livers of NASH patients. Recent findings on NK cell dysfunction in obesity (8–11) and the metabolic activities of NK cells (12, 13) have led to increased interest in a potential role for NK cells in NAFLD pathogenesis but the field is still in its infancy, not least because most previous studies did not distinguish between NK cells and ILC1 or liver-resident NK cells (14).

NK cells can limit fibrosis in liver diseases of various etiologies by controlling the activity of hepatic stellate cells (15, 16). In a mouse study in which a NASH-like disease was induced using a methionine and choline deficient diet, IFNγ production by NKp46^+^ NK cells and ILC1 protected against disease (17). On the other hand, NK cells seem to promote obesity, with NK cell-deficient mice gaining less weight in response to obesogenic diets (18–20) and displaying less fat accumulation and inflammation in their livers (21). There is also evidence that NK cells can be harmful in chronic inflammatory liver disease, where they contribute to pathology by killing hepatocytes (22, 23). In support of this being the case in NASH, mice lacking either TRAIL (an apoptosis-inducing TNF family ligand expressed by NK cells) or its receptor are partially protected against obesity-associated NASH (24, 25).

Here, we investigate the activity of NK cells in a mouse model of NASH and in the livers of NAFLD patients. We report that NK cells in the livers of obese mice are less able to degranulate and kill cancerous target cells than those from lean mice, a phenotype that extends to a reduced *in vivo* ability to kill cancer cells. This decrease in cytotoxicity is associated with a shift toward an ILC1-like phenotype, which seems to be at least partially mediated by high levels of TGFβ produced in the obese liver. Finally, we show that in humans, as in mice, NK cells from obese livers are less able to degranulate and kill.

## Results

### NK Cells in the Livers of Obese Mice Are Less Cytotoxic Than Those in the Livers of Lean Mice

To investigate the activity of NK cells and ILC1 in the liver during obesity-associated liver disease, we examined the spleens and livers of mice that were kept for up to 24 weeks on a high fat and sugar diet (26). As previously reported, mice on the diet became obese (Supplementary Figure 1A), accumulated fat in their livers (Supplementary Figure 1B) and displayed dysregulated glucose homeostasis (Supplementary Figure 1C). Mice also displayed histological evidence of NAFLD (Supplementary Figures 1D,E) and increased circulating alanine transaminase (ALT), which is an indicator of liver damage (Supplementary Figure 1F).

**Figure 1:**
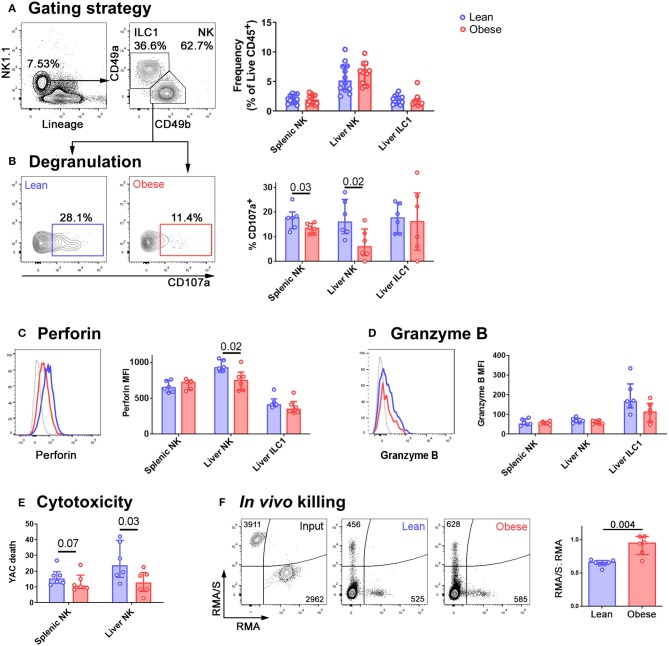
NK cells in the livers of obese mice are less cytotoxic than those in lean mice. **(A)** Immune cells were isolated from mouse livers. NK cells were identified by scatter, and as live CD45^+^ Lineage-negative NK1.1^+^ CD49b^+^ cells. ILC1 were identified as live CD45^+^ Lineage-negative NK1.1^+^ CD49a^+^ cells. The frequency of NK cells and ILC1 as a percentage of live CD45^+^ cells in the spleens and livers of lean and obese mice is shown. *n* = 12 mice per group, medians and IQRs are shown. **(B)** Intrahepatic leukocytes were cultured for 4 h in the presence of anti-CD107a and Brefeldin A. Representative CD107a staining of NK cells from a lean (left, blue) and an obese (right, red) mouse and summary data are shown. **(C)** Representative perforin staining in liver NK from a lean (blue trace) and an obese (red trace) mouse. MFI of perforin in splenic NK, liver NK, and liver ILC1 from lean and obese mice are shown. **(D)** Representative granzyme B staining in liver NK from a lean (blue trace) and an obese (red trace) mouse. Gray traces represent internal negative controls. MFI of granzyme B in splenic NK, liver NK, and liver ILC1 from lean and obese mice are shown. **(E)** NK cells were sorted from the spleens and livers of lean and obese mice and cultured with the cancerous NK cell target line YAC-1. YAC-1 death at 24 h is shown. **(F)** CTV-labeled RMA/S (NK cell targets) CTB-labeled RMA (recovery control) cells were mixed in a 1:1 ratio and intravenously injected into lean or obese mice. Representative input cells (leftmost, black) and recovered cells from lean (center, blue) and obese (rightmost, red) mice are shown. The RMA/S:RMA ratio in recovered splenocytes, normalized to input ratio, is shown. For panels **(B–F)**, *n* = 6 mice per group; significance was determined using Mann Whitney *U*-Tests; medians and IQRs are shown.

We did not observe any difference in NK cell (defined as Lineage-negative NK1.1^+^CD49a^−^CD49b^+^) or ILC1 (defined as Lineage-negative NK1.1^+^CD49a^+^CD49b^−^) frequencies in the spleens or livers of obese compared to lean mice (Figure 1A) but NK cells isolated from the livers of mice that had been kept on the obesogenic diet for 12 weeks degranulated less than those from the livers of their lean littermates (Figure 1B). This was also the case for NK cells isolated from spleens, although the reduction was smaller (a difference in the medians of 4.3% in splenic NK cells, compared to 10.0% in liver NK cells; Figure 1B). We observed no difference in the degranulation of liver ILC1 between lean and obese mice. We also found a significant reduction in the expression of perforin by NK cells in the livers of obese mice, that we did not detect in splenic NK cells (Figure 1C). This suggests that NK cells from the livers of obese mice are both less able to degranulate and less able to kill target cells than those from their lean littermates. We did not observe any difference in the expression of granzyme B (Figure 1D) although this may be accounted for by the low levels at which this protein is expressed in unstimulated mouse NK cells (27).

The reduced ability of NK cells in obese mice to degranulate suggests that their ability to kill cancerous target cells will be impaired. To determine whether this was the case, and to examine the relative defects in NK cell killing in the spleens and livers of obese compared to lean mice, we sorted NK cells from the two organs and assessed their ability to kill YAC-1 cancerous target cells *in vitro*. NK cells from both the spleens and livers of obese mice were less able to kill YAC-1 cells than those from their lean littermates, although the defect in killing was only significant (at *p* < 0.05) in NK cells isolated from the liver (Figure 1E). Similar to our observations of degranulation, the reduction in target cell killing was greater in NK cells isolated from livers than those isolated from spleens (a difference in the medians of 2.7% in splenic NK cells, compared to 10.1% in liver NK cells).

To determine whether this affects target cell killing *in vivo*, we next injected mice intravenously with equal numbers of fluorescently-labeled RMA/s cells (which are MHC class I-negative NK targets) and RMA cells (which are not NK targets and act as a recovery control) (28). We collected spleens from the recipient mice and determined the number of cells remaining in each population by flow cytometry. The lean mice had eliminated the RMA/s cells with approximately 40% efficiency. In contrast, the obese mice had eliminated them with only 5% efficiency (Figure 1F).

### Perforin-Mediated Cytotoxicity Promotes Fibrosis in NAFLD

Clearly, the diminished ability of NK cells to kill target cells in obese animals is likely to limit cancer surveillance. However, since we observed the largest reductions in both degranulation and cytotoxicity in NK cells isolated from the liver, and since the reduction in perforin expression seemed to particularly affect NK cells in the liver, we sought to define the likely effect of these changes in NAFLD. Given that TRAIL-mediated cytotoxicity exacerbates NAFLD (24, 25), we speculated that there might be some advantage to the reduced perforin-mediated cytotoxicity we observed in the obese liver. We hypothesized that perforin-mediated killing is harmful in the liver during obesity, and that the reduction in NK cell degranulation, perforin expression and cytotoxicity could therefore be protective. In this case, we would expect mice lacking perforin to suffer less severe liver disease in obesity. We therefore determined the extent of liver damage in wild type and perforin-deficient mice that had been kept on the obesogenic diet for 24 weeks. This later timepoint was chosen so that we could compare the development of fibrosis, which is not usually pronounced at 12 weeks in this model, between the two genotypes.

**Figure 2:**
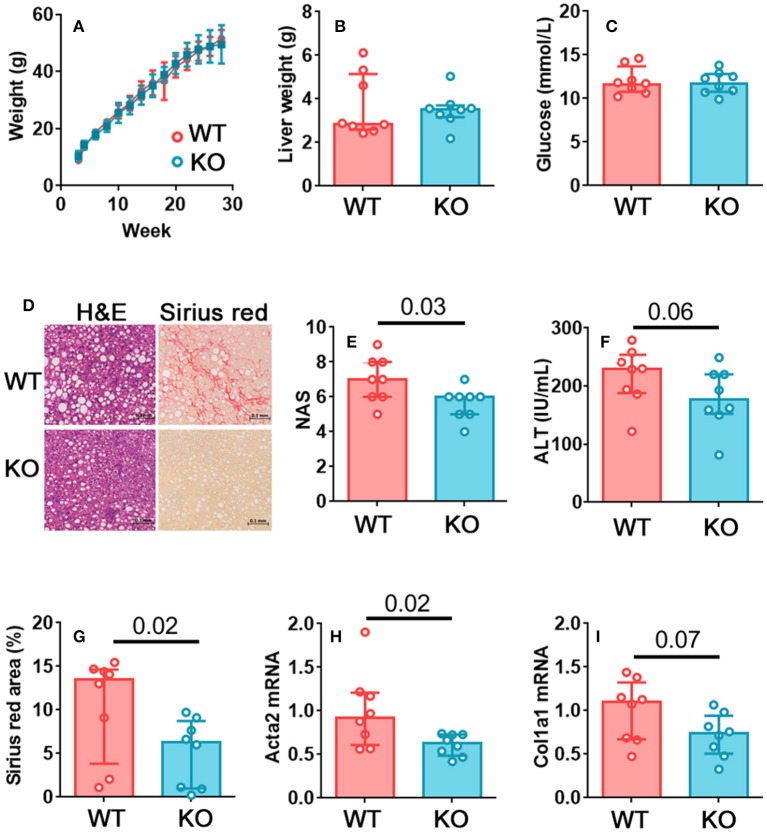
Perforin-mediated killing promotes fibrosis in NAFLD. **(A)** Growth curves for wild type and perforin knockout mice on the obesogenic diet. **(B)** Liver weights for wild type and perforin knockout mice after 24 weeks on the obesogenic diet. **(C)** Plasma glucose levels. **(D)** Representative H&E and Picrosirius red staining in a wild type and a perforin knockout mouse. **(E)** Histological score in wild type and perforin knockout mouse livers, after 24 weeks on the obesogenic diet. **(F)** Plasma ALT levels. **(G)** Picrosirius red positive area in representative histological fields. **(H,I)** Acta2 and Col1a1 mRNA in livers of wild type and perforin knockout mice, after 24 weeks on the obesogenic diet. *n* = 8 mice per group; significance was determined using Mann Whitney *U*-Tests; medians and IQRs are shown.

There was no difference in weight gain (Figure 2A), hepatomegaly (Figure 2B), or glucose homeostasis (Figure 2C) between the wild type and perforin knockout mice. Therefore, unlike mice that completely lack NK cells, perforin-deficient mice gain weight and alter their glucose homeostasis similarly to wild type mice (18–20). This suggests that any differences we observe in liver pathology are unlikely to occur as a result of less severe metabolic dysregulation in these mice. We observed a significantly lower histopathological score in the perforin-deficient mice than the wild type controls (Figures 2D,E) and decreased circulating ALT, although this was not significant at *p* < 0.05 (Figure 2F). Fibrosis, determined by Picrosirius red staining, was significantly lower in perforin-deficient mice (Figures 2D,G) and real time PCR for markers of fibrosis revealed that the livers of the perforin-deficient mice expressed less *Acta2* (α smooth muscle actin; Figure 2H) and *Col1a1* (collagen type I alpha 1; Figure 2I) than those of controls, although the difference was only significant at *p* < 0.05 for *Acta2*.

### NK Cells in the Livers of Obese Mice Display a Transcriptional Profile Characteristic of ILC1

We next sought to define the mechanism by which the reduction in NK cell cytotoxicity occurs. To achieve this, we analyzed the transcriptomes of NK cells and ILC1 in the livers of lean and obese mice by RNASeq. Raw RNAseq data and differentially expressed gene lists are available from the National Center for Biotechnology Information Gene Expression Omnibus under accession no. GSE122828; https://www.ncbi.nlm.nih.gov/geo/query/acc.cgi?acc=GSE122828.

**Figure 3:**
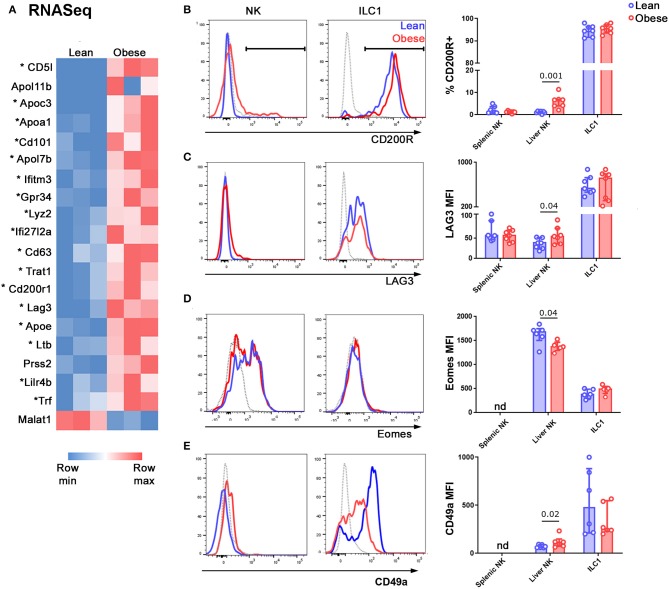
NK cells in the livers of obese mice acquire an ILC1-like transcriptional profile. **(A)** NK cells and ILC1 were sorted from the livers of lean and obese mice and examined by RNASeq. Genes that were differentially expressed by >2-fold, with padj <0.05, in NK cells of lean vs. obese mice are shown. Genes that are also differentially expressed in ILC1 compared to NK (>2-fold, with padj <0.05) are marked with an asterisk. **(B,C)** Protein expression of the inhibitory receptors CD200R1 **(B)** and LAG3 **(C)** in NK cells and ILC1 of lean (blue) compared to obese (red) mice. Gray traces represent FMO controls. **(D)** Expression of Eomes in NK cells and ILC1 of lean (blue) compared to obese (red) mice. Gray traces represent internal negative controls. **(E)** Expression of CD49a in Lineage-negative NK1.1^+^ CD49b^+^ cells (NK) and Lineage-negative NK1.1^+^ CD49b^−^ cells (ILC1) of lean (blue) compared to obese (red) mice. Gray traces represent FMO controls. *n* = 6 mice per group; significance was determined using Mann Whitney *U*-Tests; medians and IQRs are shown.

Only one gene, Malat1, was significantly (FC > 2, p_adj_ < 0.05) underexpressed in NK cells from obese mice compared to those from their lean littermates, whereas 20 genes were significantly overexpressed. Of these 20 genes, 18 were also overexpressed in ILC1 compared to NK cells (Figure 3A; genes characteristic of ILC1 are marked with an asterisk). We particularly noted that transcripts encoding the inhibitory receptors CD200R1, LAG3, and CD101, all of which are involved in limiting immune pathology in inflammatory diseases (29–32) were increased in NK cells from obese mice compared to lean, as well as in ILC1 compared to NK cells. We confirmed the increase in expression at the protein level for CD200R1 (Figure 3B) and LAG3 (Figure 3C). We also considered the possibility that a decrease in activating receptor expression could account for the decreased NK cell activity in obese mice, but we did not find any difference in NKG2D (Supplementary Figure 2A) and NKp46 (Supplementary Figure 2B) levels between NK cells from lean and obese mice.

NK cells and ILC1 express the transcription factor Tbet at roughly similar levels, although they rely on it to different extents (33–37). Consistent with this, we did not detect any change in the expression of Tbet in NK cells of the livers of lean compared to obese mice (Supplementary Figure 2C). On the other hand, the transcription factor Eomes is expressed only by NK cells and specifies the NK cell lineage (38). We observed decreased expression of Eomes in NK cells in the livers of obese compared to lean mice (Figure 3D). We also examined the expression of the ILC1-associated integrin CD49a and found a small but significant increase in its expression by NK cells in the livers of obese compared to lean animals (Figure 3E), consistent with a shift towards an ILC1-like phenotype.

### NK Cells in the Livers of Obese Mice Have an Altered Metabolic Profile

Gene Ontology analysis on this relatively short list of differentially expressed transcripts (Figure 3A) revealed that only one pathway was significantly overrepresented in the NK cells of obese mice, compared to lean littermates, and this was Triglyceride catabolic processes (*p* = 3.65 × 10^−3^). We therefore examined the cells for signs of altered immunometabolism.

**Figure 4:**
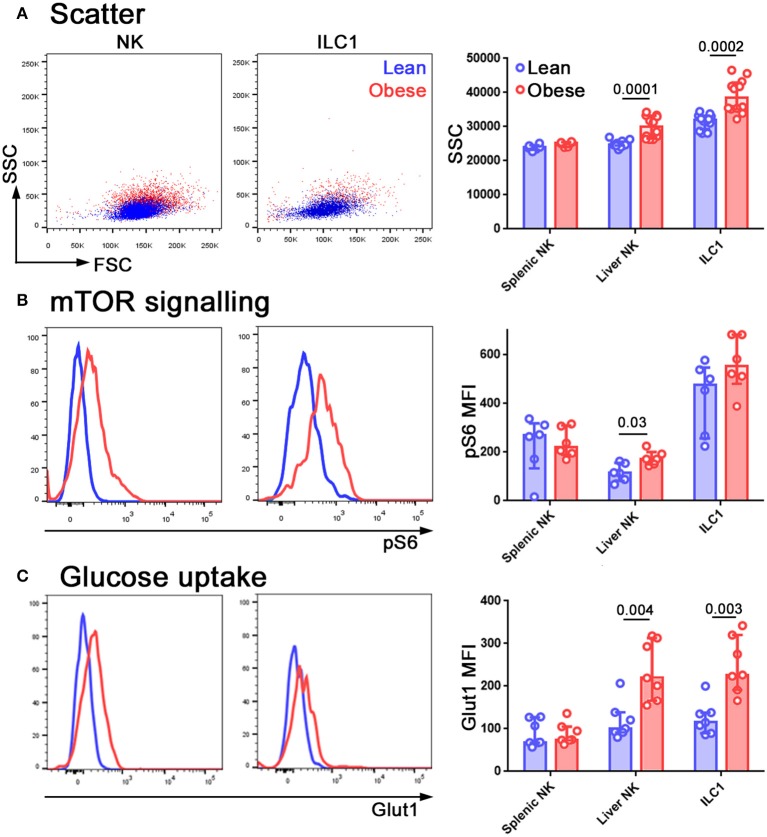
NK cells in the livers of obese mice are metabolically reprogrammed. Forward and side scatter **(A)**, pS6 staining **(B)**, and Glut1 staining **(C)** of freshly isolated NK cells and ILC1 from lean (blue) and obese (red) mice. *n* = 12 mice per group **(A)**, six mice per group **(B)** and seven mice per group **(C)**; significance was determined using Mann Whitney *U*-Tests; medians and IQRs are shown.

Scatter has been used as an indicator of metabolic alteration in NK cells (39) and we found that side scatter increased in NK cells from the livers of obese mice (Figure 4A). NK cells use mTOR for nutrient sensing and it also has an important role in immune regulation (40). The phosphorylation of ribosomal S6 protein (pS6) is used as an indicator of mTORC1 signaling and we found that pS6 was increased in NK cells freshly isolated from the livers of obese compared to lean mice (Figure 4B). Notably, in both these cases, the metabolic alterations that we observed in the NK cells served to make them more ILC1-like, similar to our findings at the transcriptional level. We also observed an increase in the expression of the glucose transporter Glut1 (Figure 4C).

### TGFβ in the Obese Liver Limits NK Cell Degranulation and Alters Their Metabolic Profile

**Figure 5:**
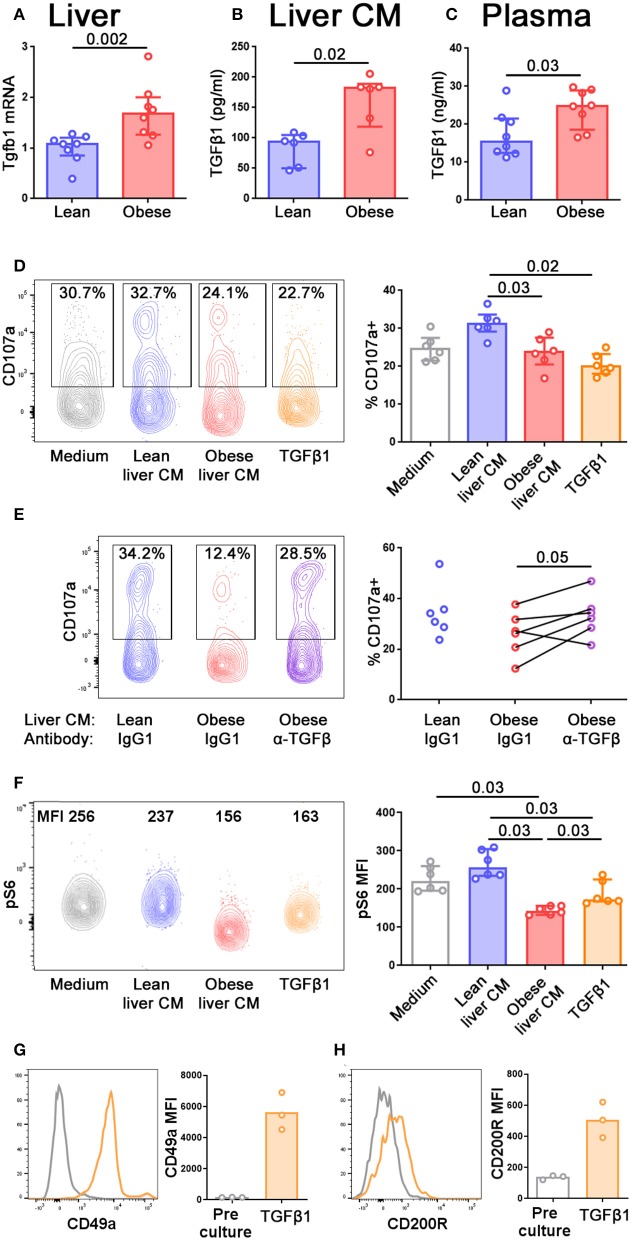
TGFβ in obese mouse livers limits NK cell degranulation and alters their metabolic profile. **(A)** Tgfb1 mRNA in the livers of lean and obese mice, normalized so that mean Tgfb1 transcript expression in the livers of lean mice = 1. **(B,C)** TGFβ1 in lean and obese liver conditioned medium **(B)** and lean and obese mouse plasma **(C)** measured by ELISA. *n* = 8 mice per group **(A,C)** and six conditioned media per group **(B)**; significance was determined using Mann Whitney *U*-Tests; medians and IQRs are shown. **(D)** Splenic NK cells were cultured for 24 h in medium alone, lean or obese liver conditioned medium, or 10 ng/ml TGFβ1. For the last 4 h of culture, CD107a antibody and Brefeldin A were added to media. *n* = 6 culture conditions per group; significance was determined using Mann Whitney *U*-Tests with Holm's correction for multiple comparisons; medians and IQRs are shown. **(E)** Splenic NK cells were cultured as described in **(D)**, with the addition of 10 μg/ml anti-TGFβ antibody or isotype control. *n* = 6 culture conditions per group; significance was determined using a Wilcoxon signed ranks test, where each pair is obese liver conditioned medium from the same mouse, with anti-TGFβ antibody or isotype control. Degranulation in NK cells cultured in lean liver conditioned medium with isotype control are shown for comparison, but were not statistically tested. **(F)** Splenic NK cells were cultured for 24 h in medium alone, lean or obese liver conditioned medium, or 10 ng/ml TGFβ1, before staining for pS6. *n* = 6 culture conditions per group; significance was determined using Mann Whitney *U*-Tests with Holm's correction for multiple comparisons; medians and IQRs are shown. **(G,H)** Splenic NK cells were cultured for 5 d in 10 ng/ml TGFβ1. CD49a **(G)** and CD200R **(H)** staining before and after culture are shown. For this small dataset, no statistical testing was carried out.

The conversion of NK cells to a less cytotoxic ILC1-like phenotype has previously been reported in a number of situations and in all these cases, TGFβ was a key cytokine driving the conversion (41–43). We therefore hypothesized that TGFβ in the obese liver might be mediating the reduction in cytotoxic ability and the acquisition of ILC1-like features that we observed. *Tgfb1* transcript was increased in the livers of obese compared to lean mice (Figure 5A) and we also observed increased TGFβ1 protein in conditioned medium from obese compared to lean livers (Figure 5B) and in the plasma of obese mice (Figure 5C).

NK cells move freely between the periphery and the liver (37). In order to define how the profile of NK cells might alter as they enter the liver, and how this might differ depending on the NAFLD status of the liver, we isolated splenic NK cells from lean animals and cultured them for 24 h in conditioned medium from either lean or obese mouse livers. We observed that the ability of NK cells to degranulate was impaired following culture in obese, compared to lean, liver conditioned medium, and we were also able to recapitulate this phenotype by culturing the NK cells in recombinant TGFβ1 (Figure 5D). Furthermore, the addition of an anti-pan TGFβ antibody to the cultures partially rescued the ability of the NK cells to degranulate following culture with obese liver conditioned medium (Figure 5E), suggesting that TGFβ in the obese liver limits the ability of NK cells to degranulate. We also examined pS6 expression as an indicator of mTORC signaling. Consistent with previous reports, NK cells cultured with TGFβ1 displayed reduced pS6 (12), as did NK cells cultured in obese liver conditioned medium (Figure 5F), suggesting that TGFβ in the obese liver can also affect the metabolic profile of NK cells.

Consistent with previous reports (42), NK cells cultured for 5 days in TGFβ1 upregulated ILC1-associated molecules such as CD49a (Figure 5G) and CD200R1 (Figure 5H). However, we did not observe this phenomenon in NK cells cultured for only 24 h in TGFβ1 and we were unable to keep NK cells alive in liver conditioned medium for 5 days. Therefore, we were unable to directly assess the ability of liver conditioned medium to convert NK cells to ILC1. Our findings that the obese liver contains high levels of TGFβ1 and that TGFβ can convert NK cells to an ILC1-like phenotype does, however, suggest that this could occur *in vivo*.

### NK Cells in the Livers of NAFLD Patients Are Less Cytotoxic Than Those From Healthy Controls

**Figure 6:**
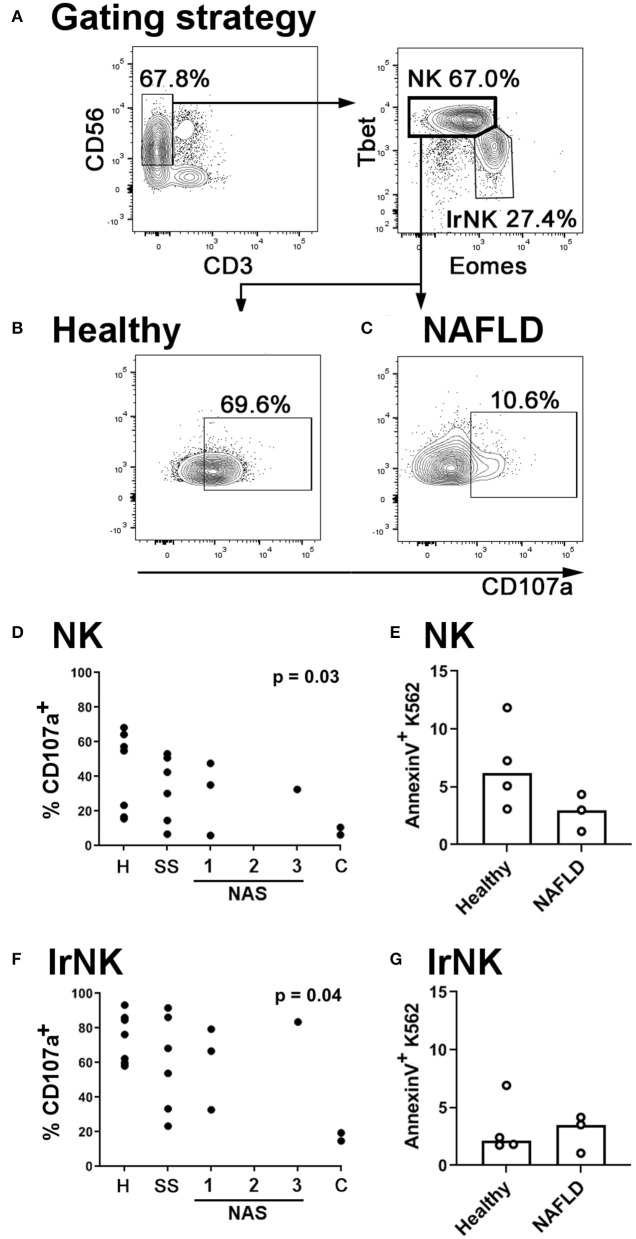
NK cells in the livers of NAFLD patients are less cytotoxic than those from healthy controls. **(A)** Immune cells were isolated from human livers. NK cells were identified by scatter, and as live CD45^+^ CD56^+^ CD3^−^ Tbet^hi^ Eomes^lo^ cells. Liver-resident NK cells (lrNK) were identified as live CD45^+^ CD56^+^ CD3^−^ Tbet^lo^ Eomes^hi^ cells. **(B,C)** Intrahepatic leukocytes were cultured for 4 h in the presence of anti-CD107a and Brefeldin A. Representative CD107a staining of NK cells from a healthy **(B)** and a NAFLD **(C)** liver is shown. **(D)** Inverse correlation between NK cell degranulation and histological score in human livers. H, healthy; SS, simple steatosis; NAS, NASH Activity Score; C, cirrhosis. Significance was determined using Spearman's Rank Correlation. **(E)** CD56^+^ CD3^−^ CD16^+^ CXCR6^−^ NK cells were sorted from healthy (*n* = 4) and NAFLD (*n* = 3) livers and cultured for 4 h with the human NK target cell, K562. At the end of culture, target cell death was assessed using AnnexinV staining. **(F)** Inverse correlation between liver-resident NK cell degranulation and histological score in human livers. Significance was determined using Spearman's Rank Correlation. **(G)** CD56^+^ CD3^−^ CXCR6^+^ liver-resident NK cells were sorted from healthy (*n* = 4) and NAFLD (*n* = 3) livers and cultured for 4 h with the human NK target cell, K562. At the end of culture, target cell death was assessed using AnnexinV staining. For **(E,G)**, medians are shown. For these small datasets, no statistical testing was carried out.

Our observations in mice prompted us to ask whether NK cells in the livers of NAFLD and NASH patients are also less able to degranulate than those from healthy controls. We isolated intrahepatic leukocytes from seven healthy livers, six livers with simple steatosis (livers that showed steatosis but no inflammation) and six livers displaying various levels of inflammation and fibrosis in addition to steatosis. We found the ability of NK cells [defined as CD3^−^CD56^+^Tbet^hi^ Eomes^lo^; (41); Figure 6A] to degranulate was inversely correlated with disease severity (Figures 6B–D). In seven samples where we were also able to sort the NK cells and assess their ability to kill the classical human NK cell target line K562, we found decreased killing by NK cells from NAFLD livers compared to healthy controls, although we were not able to collect enough data points to robustly assess the statistical significance of this relationship (Figure 6E). Interestingly, we found that the ability of liver-resident NK cells to degranulate was also inversely correlated with disease severity, although less significantly than that of circulating NK cells (Figure 6F). However, in contrast to the situation for circulating NK cells, we did not observe decreased cytotoxicity in liver-resident NK cells from NAFLD patients, compared to healthy controls (Figure 6G). This is in line with our previous observation that degranulation is not particularly correlated with cytotoxicity in human liver-resident NK cells (41).

## Discussion

It is becoming increasingly apparent that NK cells are dysfunctional in obesity and that this could be one mechanism that accounts for the well-established link between obesity and cancer (8–11). These studies have largely focused on NK cells in the blood, but in this study we looked at how NK cells in the liver change during obesity and the impact that this may have not only on cancer immunosurveillance, but also on NAFLD pathogenesis.

We found that in obesity NK cells in the livers of both humans and mice are less able to degranulate and kill target cells. This is consistent with reports that NK cells taken from the blood of obese humans (8–10, 44) or the spleens of obese mice (10) are less cytotoxic than those taken from their lean counterparts. On the other hand, one recent study did not find a reduction in NK cell degranulation in the blood of NAFLD patients compared to controls (7). This study also found no association between NK cell phenotype in the liver and NAFLD severity, although it did not examine degranulation or cytotoxicity. In the light of our findings in mice, that the obesity-associated defect in NK cell degranulation and killing is more marked in the liver than the periphery, one possibility is that a defect in NK cytotoxicity does exist in this cohort but, as we report here, it is necessary to look in the liver to find it.

We further show that the NK cells of obese mice are less able to kill cancerous target cells *in vivo*. It has recently been reported that NK cells that have been cultured in fatty acids before being adoptively transferred into a B16 melanoma-bearing host are less able to control the cancer than control NK cells. Obese mice are also less able to control B16 metastases than their lean counterparts, a phenotype that was associated with reduced NK cell infiltration into metastatic foci (10). Our finding that obese mice are less able to control RMA/S cells confirms these findings using a second type of cancerous target.

This reduction in NK cell cytotoxicity in obesity is detrimental to tumor immunosurveillance, but one key question is whether it is helpful or harmful in the context of NAFLD. We observed greater reductions in degranulation, perforin expression, and cytotoxicity in the liver than in the spleen. Further, TRAIL-mediated cytotoxicity is known to promote pathogenesis in NAFLD (24, 25). Therefore, we postulated that the decrease in degranulation and perforin-mediated cytotoxicity that we observed in the obese liver might protect against liver disease. In support of this idea, perforin-deficient mice suffered from less severe NAFLD. Since these animals lack perforin globally, we cannot say with certainty that the protection is not mediated at least in part by defective T cell cytotoxicity. Indeed if, as we suggest, the liver is acting to protect itself against immunopathology, it is likely that T cell cytotoxicity will also be reduced. Nevertheless, the protection from liver disease that we find in the absence of perforin does suggest that the defect in NK cell degranulation we observe in the obese liver may act to protect it.

We found the genes differentially expressed between NK cells from lean vs. obese livers to be strikingly similar to those that are differentially expressed between NK cells and ILC1, suggesting NK cell conversion to an ILC1-like phenotype in the obese liver. In support of this idea, we found the ILC1-associated proteins CD200R1 (45), LAG3, and CD49a increased in NK cells from obese livers, and expression of the NK cell lineage-defining transcription factor Eomes decreased. NK cells in the livers of obese mice also displayed an altered metabolic profile, which was somewhat like that of ILC1.

There are a number of situations in which NK cells are converted to resident or ILC1-like cells and in some of these, notably in the tumor microenvironment, this is associated with a decrease in cytotoxicity similar to our findings in the obese liver (41–43). NK to ILC1 conversion in all these situations is mediated by TGFβ. Further, TGFβ is known to alter the metabolic profile of NK cells and limit their cytotoxicity (8, 13). Therefore, we postulated that NK cell acquisition of an ILC1-like phenotype in the obese liver might be mediated by TGFβ. TGFβ levels were higher in obese than lean livers, consistent with previous reports in both mice and humans (46–48). Conditioned medium from obese livers limited the ability of NK cells to degranulate, mirroring the phenotype observed in the obese liver, and this effect was TGFβ-dependent. We also assessed the effect of liver conditioned medium on mTORC1 signaling, as determined by pS6 levels, and again observed obese liver conditioned medium mimicking the effect of TGFβ (12).

Interestingly, we observed reduced pS6 levels on short term culture with either obese liver conditioned medium or TGFβ but increased pS6 levels in the livers of mice that had been kept on the obesogenic diet for 6 months. It has previously been suggested that there may be differences in the kinetics of altered mTORC1 signaling in short-term vs. longer-term TGFβ exposure, and this may be at play here (13). In support of this idea, the short- and long-term effects of obesity on NK cell metabolism are known to differ: in a pediatric cohort, obesity was associated with increased Glut1 and pS6 expression (9), whereas in adults, obesity was associated with decreased 2-NBDG uptake (a proxy for glucose uptake) and pS6 expression (10). It is also likely to be the case that other factors present in the obese liver, for example high levels of fatty acids, affect NK cell cytotoxicity and metabolism (10), and the effects of these may interact with TGFβ, possibly in a manner which changes over time. This could also explain our observation that obese liver conditioned medium depresses NK cell pS6 expression more than TGFβ alone.

In this study, we have shown that NK cells in the liver display a less cytotoxic ILC1-like phenotype in the context of obesity and that this is likely to be at least partially TGFβ-mediated. Our finding that perforin-mediated killing is harmful in NAFLD suggests that this reduction in NK cell cytotoxicity is protective in the liver, albeit at the cost of increased susceptibility to cancer. As well as mediating conversion of NK cells to ILC1 (41–43), TGFβ also alters NK cell metabolism and cytotoxicity (8, 13). It has recently been suggested that NK cell metabolism could be targeted to prevent cancer (40). In the light of recent findings that NK cells are less cytotoxic in obesity and that this is at least partially mediated by metabolic changes (8–10, 44), it might be tempting to consider using such treatments as a way to break the well-established link between cancer and obesity (49). However, the implication that reduced NK cell cytotoxicity may protect the liver during obesity sounds a note of caution about such approaches, which may result in adverse effects in the liver. Indeed, NK cell-derived ILC1-like cells play a role in tissue repair (42) so the possibility that the ILC1-like cells we see emerging from NK cells in the obese liver may even have a reparative effect is worthy of further investigation.

## Materials and Methods

### Human Liver Biopsies

Liver biopsies were taken from livers that were destined for transplantation, but that were discarded because of long warm ischemic time, vascular abnormalities, or tumors elsewhere in the patient (*n* = 7), or because they displayed signs of NAFLD or NASH on histopathologic examination (*n* = 10). Biopsies were also taken from livers explanted during transplantation for NASH (*n* = 2). Intrahepatic leukocytes were isolated as previously described (41). Briefly, liver tissue was finely minced using scalpels, passed through a 70-μm strainer, and the collected cells were layered onto Ficoll (GE Healthcare, Amersham, U.K), centrifuged (400 × g, 20 min, 20°C, light braking), and the interface was taken and washed twice with PBS (750 × g, 15 min, 20°C). Ethical approval for use of human liver samples was obtained through the Royal Free Hospital Biobank (National Health Service Research Ethics Committee approval no. 11/WA/0077, study no. 9455).

### Mice

Female C57BL/6J (bred in house) or Prf1^−/−^ mice (Charles River; RRID:MGI:5576721) were randomized at weaning onto standard chow (RM1) or a highly palatable obesogenic diet consisting of 22.6% fat, 23.0% protein, and 40.2% carbohydrate (w/w); diet code 824018—“45% AFE fat” Special Dietary Services, Essex, UK), supplemented with sweetened condensed milk (Nestle) *ad libitum* (26).

After 12 or 24 weeks on the diet, mice were sacrificed by direct cervical dislocation. Blood was collected by cardiac puncture and centrifuged at 10,000 × g for 10 min at room temperature to isolate serum for ALT and glucose measurement and pieces of liver were fixed in 10% neutral buffered formalin, fixed, sectioned, and stained with H& E or Picrosirius red. Sections were blinded and scored by a hepatopathologist, using the Brunt-Kleiner NASH activity score [NAS; (50)]. Spleens were collected and splenocytes isolated as previously described (51). Intrahepatic lymphocytes were isolated using an adaptation of the method from Cuff and Male (51). Briefly, finely minced liver tissue was collected in RPMI 1640 medium (Life Technologies brand; Thermo Fisher Scientific, Hudson, NH) and passed through a 70 μm cell strainer. The suspension was spun down (500 × g, 4°C, 10 min) and the pellet resuspended in RPMI 1640 medium. The cell suspension was layered over 24% Optiprep (Sigma-Aldrich) and centrifuged without braking (700 × g, RT, 20 min). The interface layer was taken and washed in HBSS without Ca^2+^ Mg^2+^ (Lonza, distributed by VWR, Lutterworth, UK) supplemented with 0.25% bovine serum albumin (Sigma-Aldrich, Hammerhill, U.K) and 0.001% DNase I (Roche, distributed by Sigma-Aldrich).

Animal husbandry and experimental procedures were performed according to UK Home Office regulations and institute guidelines, under project license 70/8530.

### *In vivo* NK Cytotoxicity Assays

RMA and RMA/s thymoma cells (cultured in Iscove's modified Dulbecco's medium supplemented with 10% FCS; Life Technologies) were labeled with Cell Trace Blue or Cell Trace Violet, respectively, according to the manufacturer's instructions (Thermo Fisher Scientific). 10^7^ cells of each cell type were injected intravenously into control or obese mice. After 4 h, recipients were sacrificed by direct cervical dislocation and the spleens were examined for fluorescent target cells.

### *Ex vivo* NK Cell Activity Assays

For degranulation assays using mouse NK cells, total intrahepatic leukocytes or splenocytes were cultured for 4 h at 10^7^ cells/mL in RPMI 1640 medium supplemented with 10% FCS, 25 mM HEPES, 1 mM sodium pyruvate, 50 μM 2-ME, MEM nonessential amino acids, penicillin, and streptomycin (all Life Technologies brand; Thermo Fisher Scientific, Waltham, MA, USA). Brefeldin A (1 μg/mL; Sigma-Aldrich) and anti-CD107a (1:100, eBioscience, San Diego, CA) were added to all conditions. For cytotoxicity assays, 15,000 sorted NK cells were cultured with 75,000 YAC-1 target cells in 100 μL RPMI supplemented as before for 24 h. Cell death was measured using an LDH cytotoxicity assay (Abcam, Cambridge, U.K.), according to the manufacturer's instructions. Readings were normalized between the medium only (0% cell death) and lysis buffer (100% cell death) controls.

Degranulation and cytotoxicity assays with human NK cells were carried out as previously described (41). Briefly, freshly isolated intrahepatic lymphocytes were cultured for 4 h with Brefeldin A (10 μg/ml; Sigma-Aldrich), Monensin (2 μM; Sigma-Aldrich), and 5 ng/ml PerCP–eFluor 710-conjugated anti-human CD107a (clone eBioH4A3; eBioscience). Cell surface staining was performed at the end of the assay. For cytotoxicity assays, sorted cells were cultured with K562 for 4 h at a 1:1 ratio in 50 μl of RPMI 1640 medium supplemented as above. At the end of the assay, target cell death was assessed using Annexin V-FITC (BD Biosciences) and propidium iodide.

### Cell Culture Experiments

Liver conditioned medium was produced by culturing 500 mg of finely minced liver tissue in serum-free M199 medium (Gibco brand, Thermo Fisher Scientific) for 48 h. The medium was cleared of debris by being passed through a 70 μm strainer and by centrifugation at 500 × g for 5 min at 4°C. Splenic NK cells were cultured for 24 h in RPMI supplemented as before mixed 3:1 with liver conditioned medium or unconditioned M199 medium. TGFβ1 (10 ng/mL, PeproTech, Rocky Hill, NJ), anti-mouse TGFβ (10 μg/mL, clone 1D11 R&D Systems, Minneapolis, MN) or isotype control antibody (10 μg/mL, clone 11711 R&D Systems) were added as indicated.

### Flow Cytometry

Details of antibodies used in the study are given in Table 1. The lineage cocktail for mouse cells consisted of CD3, CD8α, CD19, and Gr1. Dead cells were excluded using fixable viability dye eFluor 450 (eBioscience) (4°C, 15 min). Surface staining was carried out in PBS supplemented with 1% FCS (4°C, 15 min). Intracellular staining was carried out using Human FoxP3 Buffer (BD Biosciences, Oxford, UK), except for pS6 staining, which was carried out using Cytofix/cytoperm (BD Biosciences). Data were acquired on an LSRFortessa II (BD Biosciences) and analyzed using FlowJo v.X.0.7 (Tree Star, Ashland, OR, USA). Sorting was carried out on an Aria (BD Biosciences).

**Table 1:** Details of antibodies used in the study.

**Antibody**	**Clone**	**Fluorophore**	**Dil**	**Host animal**	**Manufacturer**	**Catalog #**	**RRID**
Anti-human CD3	SK7	APC-eFluor 780	1/200	Mouse	eBioscience	47-0036-42	AB_10717514
Anti-human CD45	HI30	PE	1/100	Mouse	eBioscience	12-0459	AB_1944374
Anti-human CD56	NCAM16.2	Brilliant Violet 510	1/200	Mouse	BD Biosciences	563041	AB_2732786
Anti-human CD107	eBioH4A3	PerCP-eFluor 710	1/100	Mouse	eBioscience	46-1079-42	AB_2573706
Anti-human Eomes	WD1928	PE-eFluor 610	1/100	Mouse	eBioscience	61-4877-42	AB_2574616
Anti-human/mouse Tbet	eBio4B10	PE-Cy7	1/100	Mouse	eBioscience	25-5825-82	AB_11042699
Anti-mouse CD3	17A2	FITC	1/200	Rat	Biolegend	100203	AB_312660
Anti-mouse CD3	17A2	PerCP-Cy5.5	1/200	Rat	Biolegend	100218	AB_1595492
Anti-mouse CD8α	53-6.7	FITC	1/200	Rat	Biolegend	100705	AB_312744
Anti-mouse CD8α	53-6.7	PerCP-Cy5.5	1/200	Rat	Biolegend	100734	AB_2075238
Anti-mouse CD19	6D5	FITC	1/200	Rat	Biolegend	115505	AB_313640
Anti-mouse CD19	6D5	PerCP-Cy5.5	1/200	Rat	Biolegend	115534	AB_2072925
Anti-mouse CD45	30-F11	Brilliant Violet 510	1/200	Rat	Biolegend	103137	AB_2561392
Anti-mouse/rat CD49a	Ha31/8	Alexa Fluor 647	1/100	Armenian hampster	BD Biosciences	562113	AB_11153312
Anti-mouse/rat CD49a	Ha31/8	BUV 395	1/200	Armenian hampster	BD Biosciences	740262	AB_2740005
Anti-mouse CD49b	DX5	PerCP-eFluor 710	1/200	Rat	eBioscience	46-5971	AB_11149865
Anti-mouse CD49b	DX5	PE-eFluor 610	1/200	Rat	eBioscience	61-5971-80	AB_2574645
Anti-mouse CD107a	1D4B	PE-Cy7	1/100	Rat	Biolegend	121620	AB_2562147
Anti-mouse CD200R1	OX-110	PE	1/50	Rat	BioLegend	123907	AB_2074081
Anti-mouse Eomes	Dan11mag	PE-Cy7	1/100	Rat	eBioscience	25-4875	AB_2573453
Anti-mouse/rat/human Glut1	EPR3915	Alexa Fluor 647	1/100	Rabbit	abcam	ab195020	AB_2783877
Anti-mouse Gr-1	RB6-8C5	FITC	1/200	Rat	Biolegend	108405	AB_313370
Anti-mouse Gr-1	RB6-8C5	PerCP-Cy5.5	1/200	Rat	Biolegend	108428	AB_893558
Anti-mouse Lag3	C9B7W	PE/Dazzle 594	1/100	Rat	Biolegend	125223	AB_2572081
Anti-mouse NK1.1	PK136	APC-eFluor 780	1/100	Mouse	eBioscience	47-5941	AB_10853969
Anti-mouse Perforin	S16009B	PE	1/50	Rat	Biolegend	154405	AB_2721640
Anti-mouse pS6	D57.2.2E	APC	1/50	Rabbit	Cell Signaling	4858	AB_916156

### Real Time PCR

Liver sections were dissected directly into RNAlater and RNA was extracted using an RNeasy Lipid Tissue Mini kit (both from Qiagen, Manchester, U.K.). cDNA was made using a Transcriptor First Strand cDNA Synthesis kit (Roche, Welwyn Garden City, U.K.) and real-time PCR was performed using TaqMan (Applied Biosystems, Warrington, U.K.) primer/probe sets recognizing Hprt1 (Mm00446968_m1), Acta2 (Mm01546133_m1), Col1a1 (Mm00801666_g1), and Tgfb1 (Mm01178820_m1).

### RNASeq

Total RNA was extracted from sorted cells using an RNeasy Micro kit (Qiagen) and libraries were prepared from 2 ng of total RNA using the NEBNext low input kit (New England Biolabs, Hitchen, U.K.). Libraries were assessed for correct size distribution on the Agilent 2200 TapeStation and quantified by Qubit DNA High Sensitivity assay (Thermo Fisher Scientific) before being pooled at an equimolar concentration. Samples were sequenced on a NextSeq 500 (Illumina, Essex, U.K). Differential expression analysis was carried out using SARTools (52), filtering at padj <0.05 and FC > 2.

### TGFβ1 ELISA

Total TGFβ1 protein concentrations in plasma and liver conditioned medium were determined using a mouse TGF-beta1 DuoSet ELISA (R&D Systems), according to the manufacturer's instructions.

### Statistics

The data was analyzed and found not to be normally distributed. Significance was therefore determined using non-parametric tests: Mann Whitney *U*-Tests (for unpaired data), Wilcoxon signed rank tests (for paired data) or Spearman's tests (for correlations) as indicated in the figure legends. *P*-values < 0.05 are reported. Bars represent medians and interquartile ranges; individual data points are also plotted.

## Data Availability

Raw RNASeq data and differentially expressed genes are available from the National Center for Biotechnology Information Gene Expression Omnibus under accession no. GSE122828; https://www.ncbi.nlm.nih.gov/geo/query/acc.cgi?acc=GSE122828. All other data is available at doi: 10.17605/OSF.IO/7H4NJ.

## Ethics Statement

The studies involving human participants were reviewed and approved by National Health Service Research Ethics Committee approval no. 11/WA/0077, study no. 9455. The patients/participants provided their written informed consent to participate in this study. The animal study was reviewed and approved by UK Home Office Project License 70/8530.

## Author Contributions

VM designed the study and secured funding. AC, FS, SD, AH, TL, and VM carried out experiments. AC and VM acquired data and wrote the manuscript. RC and VM analyzed data. All authors read and approved the manuscript.

### Conflict of Interest Statement

The authors declare that the research was conducted in the absence of any commercial or financial relationships that could be construed as a potential conflict of interest.

## Supplementary Material

Supplementary Figure 1 Mice fed an obesogenic diet develop fatty liver disease. **(A)** Growth curves for mice fed an obesogenic diet (red) or standard chow (blue). **(B)** Liver weights at 3 (*n* = 12 mice/group) or 6 months (*n* = 8 mice in the lean and seven mice in the obese group). **(C)** Plasma glucose levels (*n* = 8 mice per group, except for obese at 6 months where *n* = 7 mice). **(D)** H&E and Picrosirius red staining in representative livers after 3 months on the obesogenic or standard diet. **(E)** Histological scores (*n* = 12 mice per group at 3 months, eight lean mice at 6 months and seven obese mice at 6 months). **(F)** Plasma ALT levels (*n* = 12 mice per group at 3 months, eight lean mice at 6 months and seven obese mice at 6 months). Significance was determined using Mann Whitney *U*-Tests; medians and IQRs are shown.

Supplementary Figure 2 No difference in NKG2D, NKp46, or Tbet expression in NK cells from lean vs. obese mice. NKG2D **(A)**, NKp46 **(B)**, and Tbet staining **(C)** of freshly isolated NK cells and ILC1 from lean (blue) and obese (red) mice. Gray traces represent internal negative controls **(A–C)**. *n* = 6 mice per group; significance was determined using Mann Whitney *U*-Tests; medians and IQRs are shown.
