# Supplementary figures and images for: The Obese Liver Environment Mediates Conversion of NK Cells to a Less Cytotoxic ILC1-Like Phenotype
Source: Front Immunol. 2019 Sep 11;10:2180. doi: 10.3389/fimmu.2019.02180 (PMC6749082; doi:10.3389/fimmu.2019.02180)

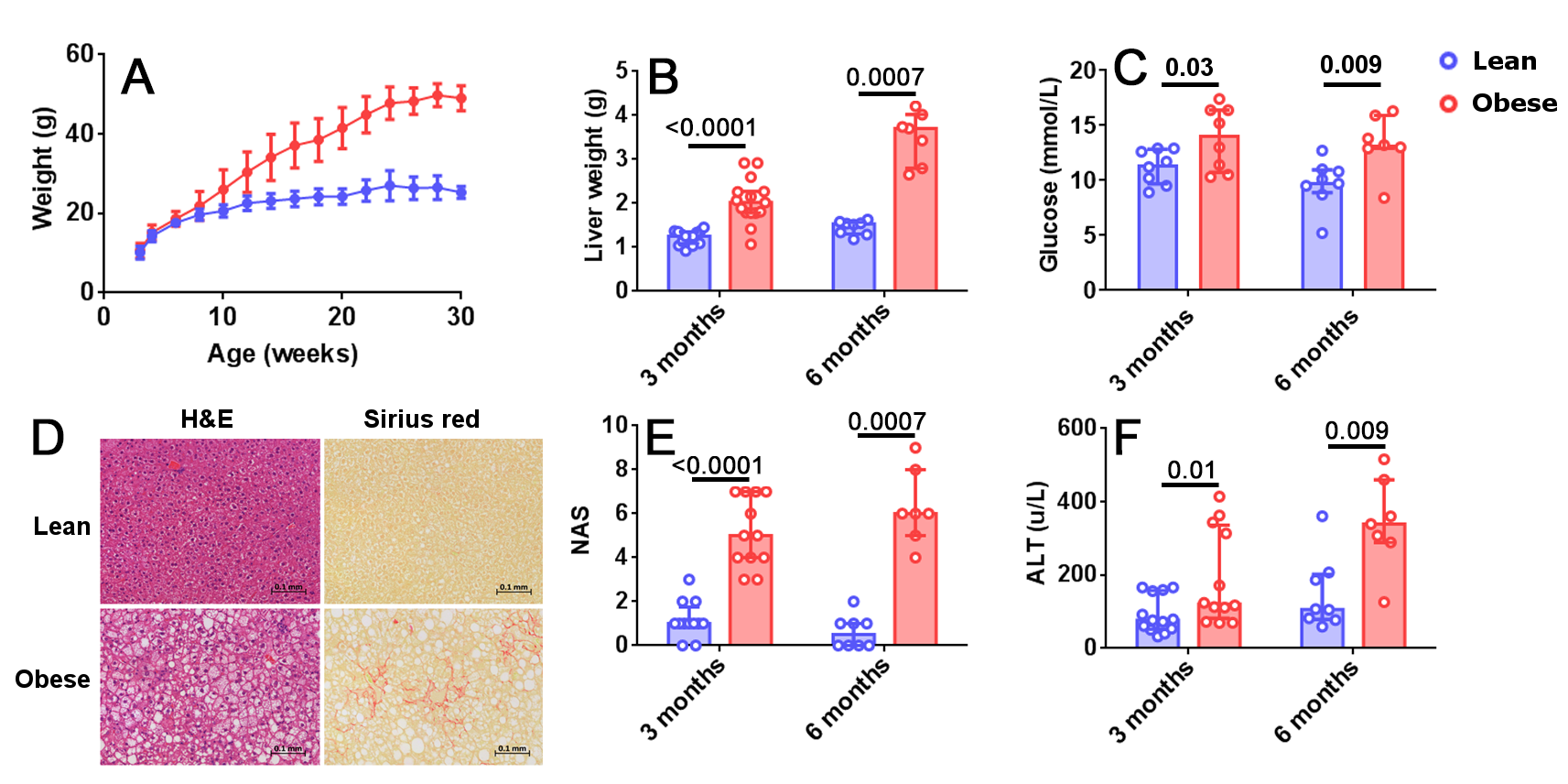

Supplement: Supplementary Figure 1 — Mice fed an obesogenic diet develop fatty liver disease. (A) Growth curves for mice fed an obesogenic diet (red) or standard chow (blue). (B) Liver weights at 3 (n = 12 mice/group) or 6 months (n = 8 mice in the lean and seven mice in the obese group). (C) Plasma glucose levels (n = 8 mice per group, except for obese at 6 months where n = 7 mice). (D) H&E and Picrosirius red staining in representative livers after 3 months on the obesogenic or standard diet. (E) Histological scores (n = 12 mice per group at 3 months, eight lean mice at 6 months and seven obese mice at 6 months). (F) Plasma ALT levels (n = 12 mice per group at 3 months, eight lean mice at 6 months and seven obese mice at 6 months). Significance was determined using Mann Whitney U-Tests; medians and IQRs are shown. [file Image_1.TIF]

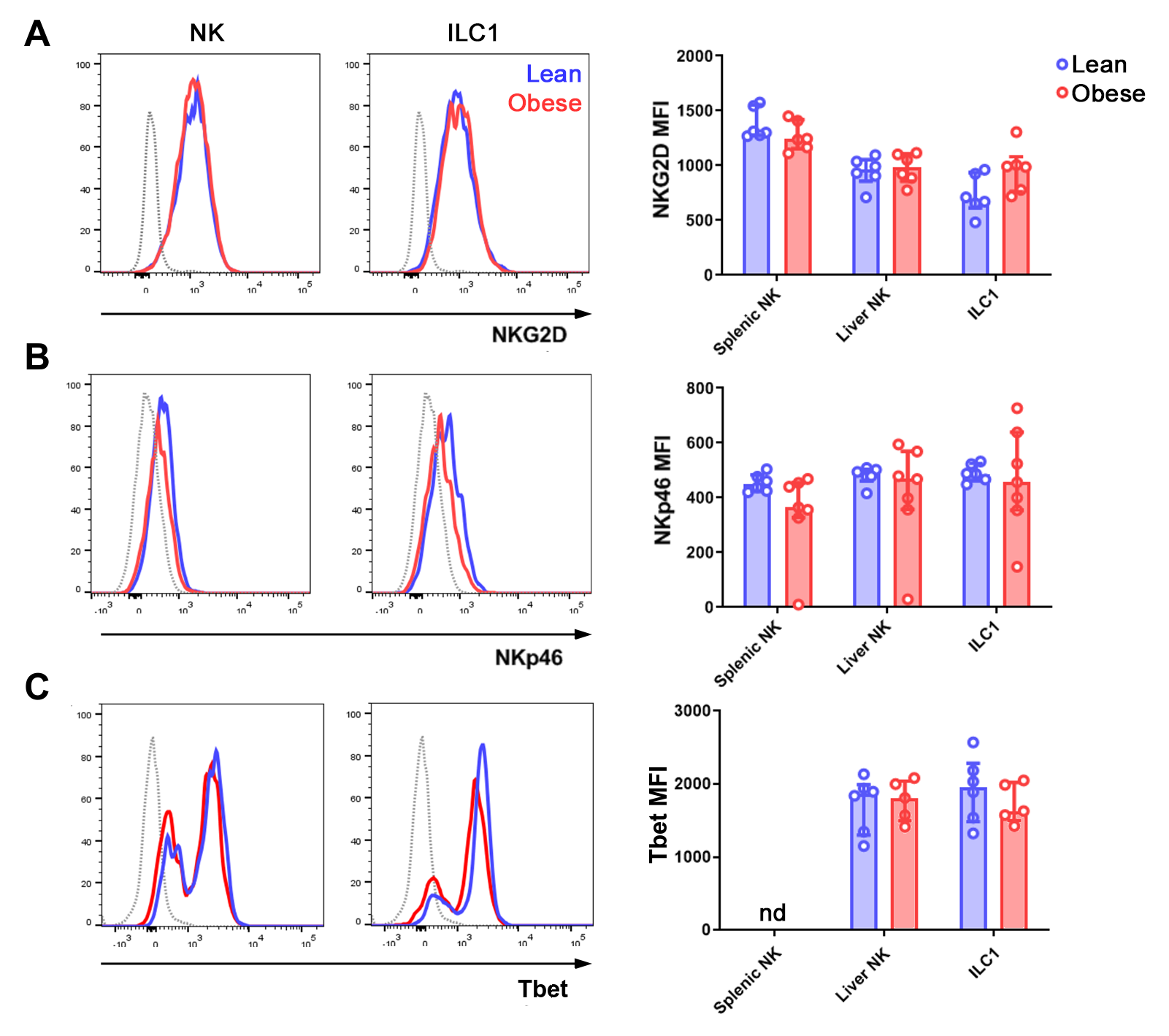

Supplement: Supplementary Figure 2 — No difference in NKG2D, NKp46, or Tbet expression in NK cells from lean vs. obese mice. NKG2D (A), NKp46 (B), and Tbet staining (C) of freshly isolated NK cells and ILC1 from lean (blue) and obese (red) mice. Gray traces represent internal negative controls (A–C). n = 6 mice per group; significance was determined using Mann Whitney U-Tests; medians and IQRs are shown. [file Image_2.TIF]
